# SneakerNet: A modular quality assurance and quality check workflow for primary genomic and metagenomic read data

**DOI:** 10.21105/joss.02334

**Published:** 2021-04-16

**Authors:** Taylor Griswold, Curtis Kapsak, Jessica C. Chen, Henk C. den Bakker, Grant Williams, Alyssa Kelley, Eshaw Vidyaprakash, Lee S. Katz

**Affiliations:** 1 Enteric Diseases Laboratory Branch (EDLB), Centers for Disease Control and Prevention, Atlanta, GA, USA; 2 Weems Design Studio, Inc., Suwanee, GA, USA; 3 Center for Food Safety, University of Georgia, Griffin, GA, USA; 4 Waterborne Disease Prevention Branch (WDPB), Centers for Disease Control and Prevention, Atlanta, GA, USA; 5 IHRC, Atlanta, GA, USA

## Abstract

Laboratories that run Whole Genome Sequencing (WGS) produce a tremendous amount of data, up to 10 gigabytes for some common instruments. There is a need to standardize the quality assurance and quality control process (QA/QC). Therefore we have created SneakerNet to automate the QA/QC for WGS.

## Statement of need

Receiving a set of primary data from whole genome sequencing or metagenomics sequencing has become commonplace and perhaps ubiquitous in bioinformatics. For a laboratory that performs WGS several times a week, some automation is necessary for both consistency and high throughput.

There are very few published workflows for performing an analysis on primary sequence data that span the breadth of initial standardized QA/QC (e.g., sequence yields, contamination checks, and subtyping). For example, the Pandoo pipeline can be given a set of genomes to run analyses: species inference, 7-gene multilocus sequence typing (MLST), resistance gene profile, plasmid profile, virulence profile, and raw read QC (Schultz et al., 2019). The Nullarbor pipeline is similar to Pandoo, but focused on public health datasets (Seemann et al., 2017). Another example is the ASA3P pipeline that runs raw read trimming, assembly, annotation, taxonomic classification, MLST, antibiotic resistance detection, virulence factor detection, reference mapping, and single nucleotide polymorphism (SNP) detection (Schwengers et al., 2019). However, no existing “broad stroke” QA/QC pipelines seem to be focused on a plugins-based architecture for batches of unrelated bacterial sequences or for batches of bacteria from different species. To that end, we have created SneakerNet. The major design principles are centered around the ability to collaboratively design plugins. With the plugins architecture, SneakerNet can dynamically change for current and future needs with input from the bioinformatics and public health community.

## Implementation

### Plugin design

SneakerNet has a modular plugin design, where the main program calls each plugin in an ordered succession. Each plugin, in turn, reads a set of genomes as input. Each plugin accepts specific flagged parameters, such that the main program can call each plugin in a standardized way. Workflows are thus defined as a specified order of plugins. An example workflow order might be genome assembly, followed by MLST, and finalized with report generation. At the time of this writing, 25 plugins are available. These plugins are listed in the documentation in a summary table, and each plugin has its own documentation page.

### Plugin development

The plugin system has drastically lowered the activation energy needed to develop a new step in a SneakerNet workflow. Documentation has been provided on how to develop a new plugin, and ‘Hello World’ plugins have been published in three different languages: Perl, Python, and Bash. Because plugins are not tied to any specific language, SneakerNet collaborators do not have to be bound by any specific language.

### Configuration

SneakerNet is highly configurable as described in the installation documentation. There are many configurations. We would like to highlight some ways that SneakerNet can be configured.

For some genera, SneakerNet comes packaged with some recommended configurations (e.g., *Salmonella* or *Legionella*), and an example genus with all options commented. These options include the minimum coverage needed for a sample to pass QC and even some detailed options to help customize a taxon for a particular plugin such as the antimicrobial resistance plugin. Therefore, a user could easily add a taxon to customize the workflow for his or her instance of SneakerNet. In fact, SneakerNet has been recently configured to accommodate the protist *Cryptosporidium* successfully with input from the CDC WDPB Molecular Epidemiology Laboratory.

Users can also customize the workflow. SneakerNet comes packaged with a default workflow which specifies the order of plugins that are run. However, if a certain analysis is not needed, e.g., 7-gene MLST, then it can be removed from the configuration. Likewise, if a new plugin is needed, it can be added into the workflow.
